# FGF21 Depletion Attenuates Colitis through Intestinal Epithelial IL-22-STAT3 Activation in Mice

**DOI:** 10.3390/nu15092086

**Published:** 2023-04-26

**Authors:** Liming Liu, Fengyuan Li, Tuo Shao, Lihua Zhang, Jiyeon Lee, Gerald Dryden, Craig J. McClain, Cuiqing Zhao, Wenke Feng

**Affiliations:** 1 College of Animal Science and Technology, Jilin Agricultural Science and Technology University, Jilin 132101, China; 2 Department of Medicine, University of Louisville, Louisville, KY 40202, USA; 3 Department of Pharmacology & Toxicology, University of Louisville, Louisville, KY 40202, USA; 4 Hepatobiology & Toxicology Center, University of Louisville, Louisville, KY 40202, USA; 5 Alcohol Research Center, University of Louisville, Louisville, KY 40202, USA; 6 Robley Rex VA Medical Center, Louisville, KY 40206, USA

**Keywords:** fibroblast growth factor 21, colitis, intestinal epithelial, IL-22, STAT3

## Abstract

Fibroblast growth factor 21 (FGF21) is a glucose and lipid metabolic regulator. Recent research revealed that FGF21 was also induced by inflammatory stimuli. Its role in inflammatory bowel disease (IBD) has not been investigated. In this study, an experimental IBD model was established in FGF21 knockout (KO) and wild-type (WT) mice by adding 2.5% (wt/vol) dextran sodium sulfate (DSS) to their drinking water for 7 days. The severity of the colitis and the inflammation of the mouse colon tissues were analyzed. In WT mice, acute DSS treatment induced an elevation in plasma FGF21 and a significant loss of body weight in a time-dependent manner. Surprisingly, the loss of body weight and the severity of the colitis induced by DSS treatment in WT mice were significantly attenuated in FGF21 KO mice. Colon and circulating pro-inflammatory factors were significantly lower in the FGF21 KO mice compared to the WT mice. As shown by BrdU staining, the FGF21 KO mice demonstrated increased colonic epithelial cell proliferation. DSS treatment reduced intestinal Paneth cell and goblet cell numbers in the WT mice, and this effect was attenuated in the FGF21 KO mice. Mechanistically, FGF21 deficiency significantly increased the signal transducer and activator of transcription (STAT)-3 activation in intestinal epithelial cells and increased the expression of IL-22. Further study showed that the expression of suppressor of cytokine signaling-2/3 (SOCS 2/3), a known feedback inhibitor of STAT3, was significantly inhibited in the DSS-treated FGF2 KO mice compared to the WT mice. We conclude that FGF21 deficiency attenuated the severity of DSS-induced acute colitis, which is likely mediated by enhancing the activation of the IL-22-STAT3 signaling pathway in intestinal epithelial cells.

## 1. Introduction

Inflammatory bowel diseases (IBDs) are associated with acute and chronic inflammatory disorders of the gastrointestinal tract and include Crohn’s disease (CD) and ulcerative colitis (UC) [1]. The prevalence of these disorders has rapidly increased due to the impact of unhealthy Western lifestyles and has now become a global health problem. Although the etiology of IBD has not been clearly specified, intensive studies have linked IBD to metabolic syndrome (MetS), which includes diabetes, obesity, and dyslipidemia [2,3], as they share some common pathophysiological features including inflammation, adipose tissue dysregulation, and gut dysbiosis. MetS treatment has been proposed as a therapeutic approach and/or prevention strategy for patients with IBD [4,5]. 

The role of adipose tissue in the development of MetS and IBD has been extensively studied [6]. In particular, our group previously demonstrated a protective role of fibroblast growth factor (FGF) 21 deficiency against adipose tissue lipolysis in a mouse model of dextran sulfate sodium (DSS)-induced colitis [7]. FGF21 is an endocrine metabolic regulator that is expressed in many metabolically active tissues such as the liver, white adipose tissue (WAT), brown adipose tissue (BAT), pancreas, and muscle [8]. FGF21 is involved in many metabolic processes and acts by binding to its receptors (FGFRs) and co-receptor beta-Klotho (KLB) [9,10]. FGF21 was first described as a glycemic regulator that stimulates glucose uptake in adipose tissue by increasing glucose transporter 1 (GLUT1) expression [11]. FGF21 transgenic mice exhibited enhanced insulin sensitivity, reduced hepatic steatosis, and increased BAT mass [12]. In contrast, FGF21-deficient mice exhibited significantly more weight gain, developed hepatic steatosis, and demonstrated adipose lipolysis disfunction when fed alcohol [13]. Recent studies found that the administration of recombinant human FGF21 ameliorated high-fat-induced hyperlipidemia and inflammation in mice [14]. 

Higher circulating levels of FGF21 have been linked to increased hepatic FGF21 synthesis [15]. Moreover, recent studies reported that FGF21 could also be induced under a variety of stress conditions, such as inflammation [16,17]. As such, subjects with obesity and non-alcoholic fatty liver disease (NAFLD) have increased systemic FGF21 levels and hepatic mRNA expression, suggesting an “FGF21 resistant” [18] status, although this remains contentious [19]. Our group and others found that levels of circulating FGF21 were higher in IBD patients and DSS-treated mice [7,20]. As FGFRs and KLB are abundantly expressed in the intestine [8], we hypothesized that FGF21 plays an important role in IBD.

Here, we demonstrated that FGF21 deficiency attenuated DSS-induced acute colitis in mice. We further showed that this protective effect of FGF21 deficiency is mediated by intestinal IL-22-induced epithelial STAT3 activation, which is crucial in combating the DSS-induced pro-inflammatory response. 

## 2. Materials and Methods

### 2.1. Mice 

FGF21 KO mice were previously described [21]. C57BL/6J were used as wild-type (WT) controls and were acquired from Jackson Laboratory (Bar Harbor, ME, USA). The mice were group-housed in an animal care facility and were exposed to a 12:12 h light–dark cycle under a controlled temperature (25 °C). All mice were provided with unrestricted access to standard mouse chow and water. All experiments were approved by the University of Louisville Institutional Review Board (No. 16.1169). 

### 2.2. DSS-Induced Colitis

At eight to ten weeks of age, male FGF21 KO and WT mice were randomly divided into four groups. The four groups were (1) WT Untreated; (2) WT DSS-Treated; (3) KO Untreated; (4) KO DSS-Treated. For the induction of acute colitis, the mice were administered DSS (2.5%, wt/vol; MW 36,000–50,000, MP Biomedicals, Solon, OH, USA) in their drinking water for 3, 5, or 7 days. The DSS solution was freshly prepared every other day. The untreated animals were provided normal water. Each experimental group comprised five to eight mice for animal investigations. Daily measurements of body weight, rectal bleeding, and stool consistency were taken. At the end of DSS administration, blood and tissue samples were collected for analyses. The disease activity index in each group was monitored daily and scored according to the previous report [22]. Experiments were repeated four times.

### 2.3. Human Subject

De-identified colon samples from patients with ulcerative colitis (UC) and healthy controls were obtained from the University of Louisville Hospital. Written informed consent and confirmation were obtained from all participants with IBD. Healthy controls were matched to subjects with IBD in terms of age, sex, and BMI. All patients recorded endoscopic confirmed diagnoses and documented the severity of UC using the Clinical Colitis Activity Index (for UC). The study was approved by the Clinical Research Ethics Committees of the University of Louisville, Louisville, KY, USA. 

### 2.4. Mouse Blood Biochemical Assays

Mouse blood samples were centrifuged at 1500× *g* for 30 min at 4 °C to obtain plasma. Following the manufacturer’s instructions, ELISA kits were used to assess the concentrations of FGF21 (R&D, Minneapolis, MN, USA). Quantitative measurements of interleukin-22 (IL-22, EMD Millipore Corp., Billerica, MA, USA), interleukin-6 (IL-6), monocyte chemotactic protein-1 (MCP-1), and tumor necrosis factor-α (TNF-α) (BD Biosciences, San Diego, CA, USA) in the mouse plasma samples were performed using ELISA kits.

### 2.5. Myeloperoxidase (MPO) Activity

Colonic tissue homogenates were used to measure MPO activity. MPO activity was evaluated using a fluorescence-based activity assay kit (Abcam, Cambridge, MA, USA), according to the manufacturer’s instructions, to calculate the MPO concentration in each sample. 

### 2.6. RNA Extraction and Quantitative Real-Time RT-PCR

Total RNA was extracted from the colon tissues with Trizol reagents (Invitrogen, Carlsbad, CA, USA). The crude RNA was then purified using lithium chloride (LiCl) as previously described [23]. For the qRT-PCR, cDNA was reverse-transcribed from the total RNA using a GenAmp RNA PCR kit (Applied Biosystems, Foster City, CA, USA). Specific primers and SYBR green PCR Master Mix (Applied Biosystems, Foster City, CA, USA) were used to amplify the cDNA. The primer sequences are listed in Table 1. The data were normalized to the 18s level for mRNA. The 2^−ΔΔCt^ method was used to calculate the relative levels of gene expression. 

### 2.7. Colon Organ Culture and Assessments of Proinflammatory Mediator

To evaluate the local levels of IL-6, interleukin-1β (IL-1β), TNF-α, and MCP-1, we generated organ cultures from untreated and DSS-treated WT and FGF21 KO mice as previously described [24,25]. In brief, the most distal 2 cm of the colon was removed and longitudinally sliced open. These strips were washed with penicillin/streptomycin (PS)-containing PBS and then further sliced into 1 cm^2^ sections, and the wet weight of each section was recorded. The colon slices were cultured in RPMI 1640 media containing PS for 24 h. An ELISA (BD Biosciences, San Diego, CA, USA) was used to test for cytokine secretion in cell-free supernatants.

### 2.8. Antibodies

Immunohistochemistry, immunofluorescence, and immunoblot analyses were performed using monoclonal antibodies against FGF21, CLCA3, cleaved caspase 3, BrdU, β-Klotho (Abcam, Cambridge, MA, USA), Lysozyme (DAKO, Carpinteria, CA, USA), E-Cadherin, phospho-Stat3, Stat3, phospho-Stat5, Stat5, phospho-Protein kinase B (Akt), Akt, Socs2, Socs3, Janus-activated kinase (Jak) 1, Bax, β-actin (Cell Signaling Technologies, Beverly, MA, USA), and β-Klotho (Santa Cruz Biotechnology, Santa Cruz, CA, USA). 

### 2.9. Isolation of Lamina Propria Lymphocytes (LPLs) 

Intestinal LPLs were isolated as previously described [26,27]. In brief, the intestinal segments were flushed with PBS, everted, and washed three times in cold PBS to remove fecal contents and mucus from the intestinal lumen. Each intestine was cut into pieces approximately 1.5 cm long and placed into conical tubes, which were incubated twice in 30 mL of IEL medium (RPMI 1640 containing PS, 2 mM EDTA, and 5% FBS) at 37 °C in a shaker at 250 rpm for 20 min each time. After the second round of shaking, the tissues were rinsed in HBSS and digested in collagenase solution (1 mg/mL Collagenase type VIII and 50 µg/mL DNase I dissolved in IEL medium) at 200 rpm for 20 min at 37 °C. The cells were filtered through a 100 µm cell strainer and fractionated using a Percoll TM (GE Healthcare) via gradient (40% Percoll on the top; 80% Percoll on the bottom) centrifugation. The LPLs were recovered at the interface of the 40 and 80% Percoll solutions.

### 2.10. Flow Cytometry 

The above-referenced collected LPLs were washed with PBS twice and gently resuspended in PBS containing 5% FBS for 15 min in the dark at 4 °C with the blocking Fc-receptor CD16/32 (2.4G2, 1:50, BD Biosciences, San Diego, CA, USA). The cells were then incubated with labeled antibodies for 30 min at 4 °C (BD Biosciences, Franklin Lakes, NJ, USA). The samples were then washed twice with PBS containing 5% FBS. The samples were immediately analyzed or fixed in PBS containing 2% paraformaldehyde. The antibodies (Abcam, Inc., Cambridge, UK) used for analysis were: FITC-conjugated anti-mouse Ly6G (1A8), Ly6C (HK1.4), or CD11b (M1/70). The cells were analyzed via flow cytometry (BD Bioscience, San Diego, CA, USA) and processed using FlowJo software (Tree Star, Ashland, OR, USA).

### 2.11. Histology

For histological analysis, the tissues were fixed in 4% paraformaldehyde, embedded in paraffin, and sectioned. The tissue sections were then stained with either Hematoxylin and Eosin (H&E) or Alcian blue. The tissue sections were photographed using a microscope, and the histopathological score was analyzed as described previously [28].

For the immunofluorescence analysis, sections were blocked with 5% bovine serum albumin (BSA) for 1 h at room temperature (RT). The sections were incubated overnight with primary antibodies at 4 °C in a humidified chamber and were subsequently incubated with Alexa Fluor 488-conjugated secondary antibodies (Invitrogen, Eugene, OR, USA). DAPI was utilized as a counterstain. Images were taken using a Zeiss LSM 510 confocal laser scanning microscope. To determine BrdU incorporation, BrdU was injected 2, 24, and 48 h before the animals were killed. 

For the immunohistochemistry assay, tissue sections were blocked as previously described. The primary antibodies were then used to specifically bind to the target protein overnight at 4 °C in a humidified chamber. The signal conversion was detected using a DAB substrate kit (DAKO, Carpinteria, CA, USA) as described in the manual. 

### 2.12. Western Blot Analysis

Tissue samples were homogenized in standard RIPA buffer with a protease and phosphatase inhibitor cocktail. Lysates were separated using 10–15% SDS-PAGE, and proteins were transferred to polyvinylidene difluoride (PVDF) membranes (Whatman, Dasel, Germany). Each membrane was blocked in PBS-T buffer containing 5% skim milk for 1 h at RT. The membrane was then probed with primary antibodies overnight at 4 °C. HRP-conjugated secondary antibodies (Cell Signaling Technologies, Beverly, MA, USA) were incubated with the membrane at RT for 1 h. Blots were imaged using the chemiluminescent imager, and Image Lab 4.1 was then used to analyze the specific bands of the images. The results are representative of three separate investigations.

### 2.13. TUNEL Assay

Cell apoptosis was detected in situ via ApopTag Peroxidase in an Apoptosis Detection Kit (Chemicon, CA, USA, USA), as per the manufacturer’s instructions [29]. In brief, sections of formalin-fixed paraffin tissue were cut at a thickness of 5 µm. The tissue sections were deparaffinized, rehydrated, and then incubated with 20 g/mL proteinase K for 15 min at RT. After being incubated with 3% hydrogen peroxide solution for 5 min, the tissue sections were treated with terminal deoxynucleotidyl transferase (TdT) for 1 h and anti-digoxigenin-peroxidase for 30 min at 37 °C. Diaminobenzidine (DAB) was then applied. Finally, the sections were counterstained with Hematoxylin. Apoptotic cells were manually counted under the microscope, where they showed as TUNEL positives as brown nuclear stains. 

### 2.14. Statistical Analysis

Data are expressed as means ± SEM. A two-way analysis of variance (ANOVA) was performed, followed by Tukey’s multiple comparisons test, for multiple comparisons of two independent variables. A one-way ANOVA, followed by Tukey’s multiple comparisons test, was performed for multiple-group comparisons, or a standard two-tailed unpaired *t*-test was used for the statistical significance analysis of groups. Statistical analyses were performed with Prism 7 (GraphPad, San Diego, CA, USA). *p* values of <0.05 were considered significant. 

## 3. Results

### 3.1. Serum FGF21 Levels Are Increased in Colitis

**Figure 1:**
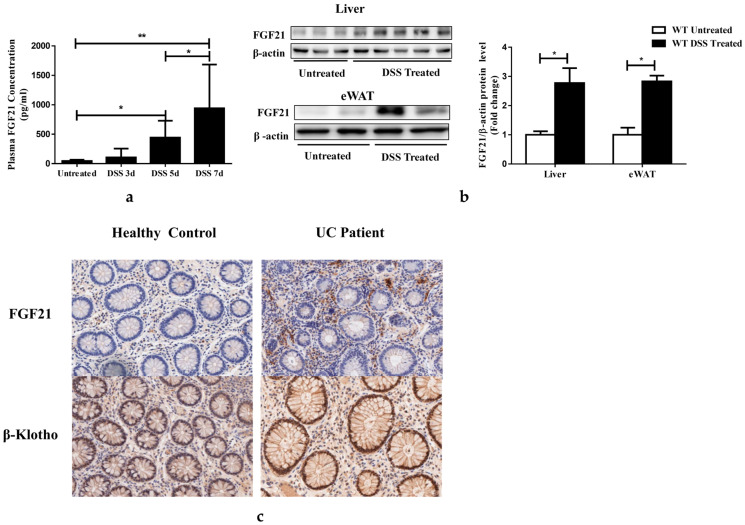
Colitis increased FGF21 expression. (**a**) Plasma FGF21 protein levels in WT mice. (**b**) Hepatic and epididymal white adipose tissue (eWAT) FGF21 protein levels, determined by immunoblots (left) and quantification (right panel). (**c**) Representative histochemistry stained images of FGF21 and β-klotho expression in biopsies from healthy control and UC patients. Data presented indicate the mean ± SEM. (**a**) Significance was determined using a one-way ANOVA followed by Tukey’s multiple comparisons test. (**b**) Significance was determined using two-tailed unpaired Student’s *t*-test (* *p* < 0.05 and ** *p* < 0.01).

Previous studies demonstrated that FGF21 was upregulated during inflammation induced by inflammatory LPSs, zymosan, and turpentine [16]. In this study, the DSS-challenged mice were established as a model of intestinal and systemic inflammation. On the fifth day of DSS administration, plasma FGF21 levels were significantly increased in DSS-treated mice and were further elevated by 21-fold on day seven (Figure 1a). The hepatic and epidydimal white adipose tissue (eWAT) expression of the FGF21 protein was increased after 7 days of DSS administration (Figure 1b), which could contribute to the elevated FGF21 plasma levels. Similar to the animal experiment, we previously showed that serum FGF21 levels were elevated in UC patients compared to the healthy controls [7]. An immunohistochemistry analysis revealed that KLB [9] protein, the unique FGF21/FGF19 co-receptor, was mainly located on the surface of the intestinal epithelium and was increased in UC patients (Figure 1c). Increased FGF21 expression was observed in the intestines of UC patients, and it was mainly located in the extra-epithelial compartments (Figure 1c) [30,31].

### 3.2. FGF21 Deficiency Protects Mice from DSS-Induced Acute Colitis

**Figure 2:**
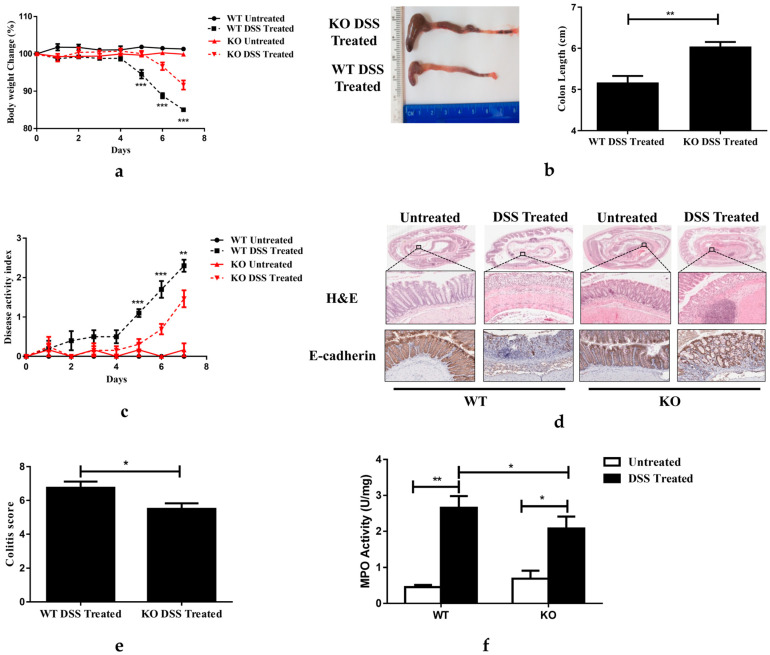
FGF21 KO mice are protected from DSS-induced acute colitis. (**a**) Bodyweight changes during the course of DSS administration. Values are presented as the percentage of untreated control mice. (**b**) Representative colon images (left panel) and colon length (right panel) in DSS-treated WT and FGF21 KO mice. (**c**) Disease activity index (DAI) scores. (**d**) Representative images of H&E-stained colon tissues (upper penal) and E-cadherin (bottom panel) immunohistochemical staining. (**e**) Colitis score. (**f**) Colon myeloperoxidase (MPO) enzymatic activity. Data presented indicate the mean ± SEM. (**a**,**c**) Statistical significance for WT DSS-Treated vs KO DSS-Treated groups was determined using a two-way ANOVA, followed by Tukey’s multiple comparisons test. (**b**,**e**) Significance was determined using two-tailed unpaired Student’s *t*-test. (**f**) Significance was determined using a two-way ANOVA, followed by Tukey’s multiple comparisons test (* *p* < 0.05, ** *p* < 0.01 and *** *p* < 0.001).

Whole-body FGF21 knockout (FGF21 KO) mice and WT control mice were analyzed to determine the role of FGF21 in the development of colitis. A seven-day DSS treatment induced symptoms of acute colitis, including substantial weight loss (Figure 2a) and colon shortening (Figure 2b) and significantly increased the disease activity index (DAI), which was calculated based on the parameters of wight loss, diarrhea, and rectal bleeding [32] (Figure 2c). The WT mice treated with DSS for seven days demonstrated increased leukocyte infiltration, crypt distortion, edema, and hemorrhage, as shown in the H&E-stained colon tissue (Figure 2d). In addition, DSS treatment led to the structural destruction of the adherens junction (AJ) through the attenuated expression of E-cadherin (Figure 2d). DSS treatment also disrupted AJ integrity via increasing the expression of E-cadherin (Figure 2d). Surprisingly, these detrimental effects of DSS treatment were attenuated in the FGF21 KO mice, as demonstrated by their reduced loss of body weight and colon shortening, decreased DAI score, and improved colon histology (Figure 2a–d). Importantly, FGF21 KO mice demonstrated an improved colitis score (Figure 2e) and reduced myeloperoxidase (MPO) activity (Figure 2f) compared to the WT mice. These results suggested that FGF21 deficiency has a protective effect on DSS-induced acute colitis.

### 3.3. FGF21 Deficiency Decreases DSS-Induced Colon Inflammation

**Figure 3:**
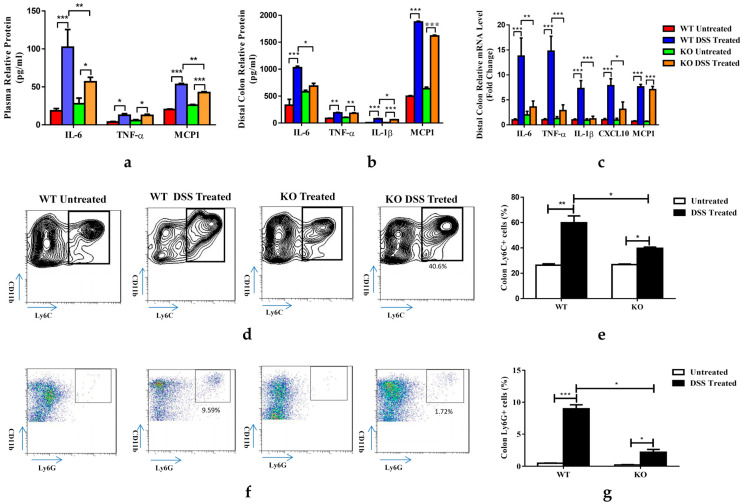
FGF21 KO mice had reduced inflammation. (**a**) Plasma levels of inflammatory mediators. (**b**) Inflammatory mediator secretion in colon tissue culture. (**c**) mRNA expression of inflammatory mediators in colon tissue. (**d**) Representative density plots via flow cytometry and (**e**) frequency expressed as percentage of CD11b+Ly6C+ myeloid cells in intestine. (**f**) Representative density plots via flow cytometry and (**g**) frequency expressed as percentage of CD11b+Ly6G+ myeloid cells in intestine. Data presented indicate the mean ± SEM. (**a**–**e**,**g**) Significance was determined using a two-way ANOVA, followed by Tukey’s multiple comparisons test (* *p* < 0.05, ** *p* < 0.01 and *** *p* < 0.001).

As colitis is a systemic inflammatory disease, we examined cytokine levels in the plasma of WT and FGF21 KO mice treated with DSS. Under untreated conditions, both genotypes of mice showed similar levels of plasma IL-6, TNF-α, and MCP-1. The seven-day DSS treatment induced a 5.7-fold increase in IL-6, a 3.4-fold increase in TNF-α, and a 2.6-fold increase in MCP-1 in the plasma of the WT mice. However, in the FGF21 KO mice, although the plasma concentrations of IL-6 and MCP-1 were increased by the DSS treatment, the extent of the increase was smaller than in the WT mice. No change in TNF-α was observed in the KO mice compared to the WT mice treated with DSS (Figure 3a). To evaluate the inflammatory status in colon tissues, we cultured distal colon explants of the mice for 24 h, and the cultural supernatants were used for a pro-inflammatory protein expression analysis. DSS treatment increased the protein levels of IL-6 (3.1-fold), TNF-α (2.2-fold), IL-1β (11-fold), and MCP-1 (3.8-fold) in the colon cultural supernatant of WT mice. Interestingly, we observed an increase in the IL-6 level in untreated KO mice compared to WT mice, and the increase via DSS treatment was decreased. Similarly, IL-1β expression was significantly reduced in the KO mice; however, compared to the WT mice, no changes were observed for TNF-α and MCP-1 (Figure 3b). Similarly, the colon mRNA expression of pro-inflammatory molecules IL-6, TNF-α, IL-1β, CXC chemokine ligand-10 (CXCL-10), and MCP-1 were markedly increased in the WT mice treated with DSS. The mRNA levels of IL-6, TNF-α, IL-1β, and CXCL-10 but not MCP-1 were reduced in the KO mice (Figure 3c). In addition, we analyzed the immune cell populations present in the colon lamina propria. An FACS analysis of the intestinal lamina propria lymphocytes (LPLs) showed that the percentages of CD11b^+^ Ly6C^+^ (Figure 3d,e) and CD11b^+^ Ly6G^+^ (Figure 3f,g) myeloid cells were decreased in the KO mice compared to the WT mice treated with DSS for 7 days. These data suggested that FGF21 deficiency alleviated DSS-induced colonic inflammation. 

### 3.4. FGF21 Deficiency Prevents DSS-Induced Reduction of Paneth and Goblet Cells 

**Figure 4:**
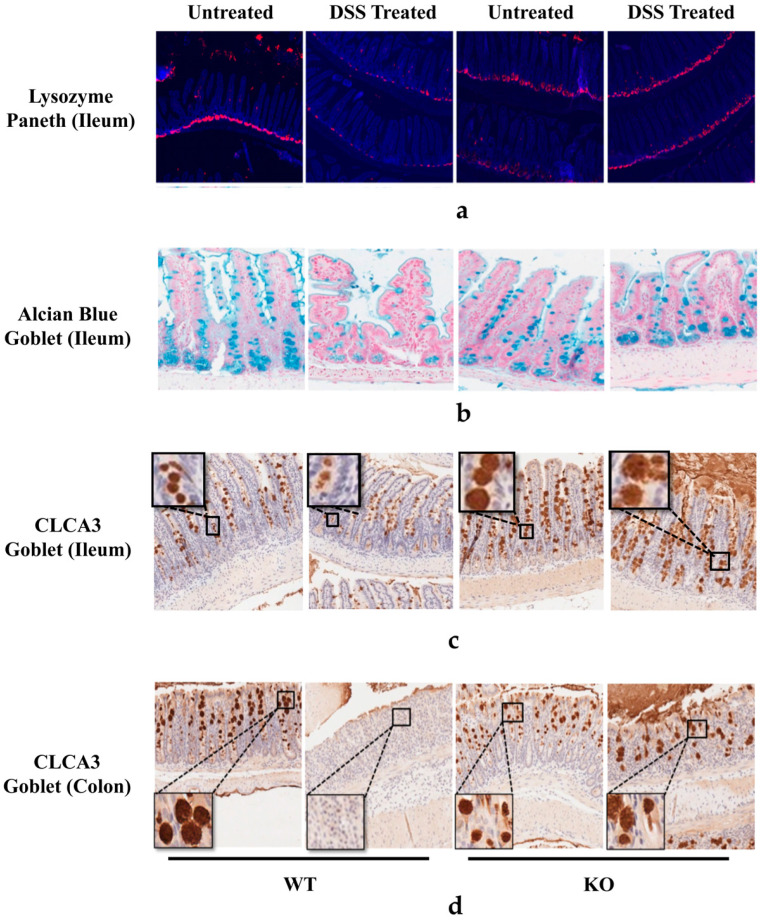
FGF21 KO mice maintain goblet and Paneth cell homeostasis. WT and FGF21 KO mice were either untreated or treated with 2.5% DSS for 7 days. (**a**) Immunofluorescence staining of Lysozymes of ileum sections in each group. (**b**) Alcian blue staining of ileum sections in each group. (**c**) Representative CLCA3 staining images of ileum and colon (**d**) sections in each group.

Paneth cells are located at the bottom of the small intestinal crypts and secrete various antimicrobial peptides which are crucial in fighting against the invasion of pathogens, modulating the commensal microbiota, and regulating innate immunity. Goblet cells produce and secrete mucins to form a protective luminal mucus layer. Both Paneth and goblet cells play a crucial role in IBD pathophysiology and were analyzed in this study. As shown in Figure 4a, the seven-day DSS treatment markedly reduced the ileal crypts’ lysozyme-positive Paneth cells in WT mice, which was not observed in the KO mice. Alcian blue (Figure 4b) and calcium-activated chloride channel 3 (CLCA3) staining (Figure 4c,d) of the ileal and colon tissues showed a loss of goblet cells and thinning of the mucus in WT DSS-treated mice, which were dramatically increased in the DSS-treated FGF21 KO mice. These data suggest that FGF21 KO protected against DSS-induced Paneth and goblet cell reduction. 

### 3.5. FGF21-Deficient Mice Were Protected against DSS-Induced Epithelial Cell Apoptosis and Impaired Proliferation 

**Figure 5:**
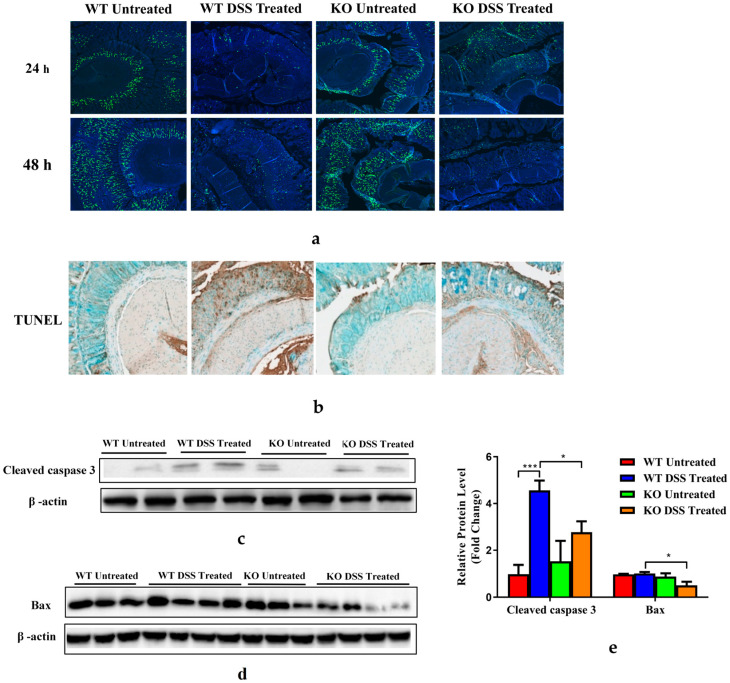
FGF21 KO mice displayed enhanced proliferation and attenuated epithelial cell death responses upon DSS treatment. Representative images of BrdU staining (**a**) and TUNEL staining (**b**) of colon tissues. Relative protein levels of apoptosis marker cleaved Caspase-3 (**c**) and Bax (**d**) in the colon tissue. (**e**) Intensity of protein bands was quantified by densitometry analysis, using β-actin levels as loading controls. Data presented indicate the mean ± SEM. (**e**) Significance was determined using two-way ANOVA, followed by Tukey’s multiple comparisons test (* *p* < 0.05 and *** *p* < 0.001).

DSS treatment causes colitis since it potentially damages the colon epithelium by directly killing intestinal epithelial cells (IECs), followed by a regenerative proliferation response aimed at restoring the epithelial barrier and limiting the propagation of colonic inflammatory responses. The ability of IECs to proliferate and regenerate is critical for restoring the integrity of the mucosal barrier [33]. To assess whether FGF21 deficiency plays an essential role in IEC proliferation, a BrdU incorporation assay was performed. After 7 days of DSS treatment, BrdU was injected intraperitoneally into mice 24 or 48 h before sacrifice. The number of BrdU positive cells was markedly decreased in the crypts of the colons in WT mice treated with DSS, but the decrease was markedly attenuated in the KO mice (Figure 5a), indicating that FGF21 deficiency restored epithelial regeneration. Similarly, a stronger BrdU staining was observed in the ileal tissues of KO mice after DSS treatment (Figure S1). DSS-induced colon apoptosis was detected by TUNEL staining in the WT mice after 7 days of DSS treatment. Fewer TUNEL-positive cells were found in the DSS-treated KO mice compared to the WT mice (Figure 5b). Immunoblotting of cleaved caspase-3 and Bax expression in colon tissues further confirmed a decreased colonic apoptotic cell death in the KO mice (Figure 5c–e). These results indicate that both the epithelial cell death and impaired regenerative proliferation caused by DSS treatment were attenuated in the FGF21 KO mice, suggesting that the decreased severity of colitis observed in the FGF21 KO mice could be attributed to decreased apoptosis and the increased proliferation of IECs.

### 3.6. FGF21 Deficiency Enhances Distal Colon STAT3 Signaling

**Figure 6:**
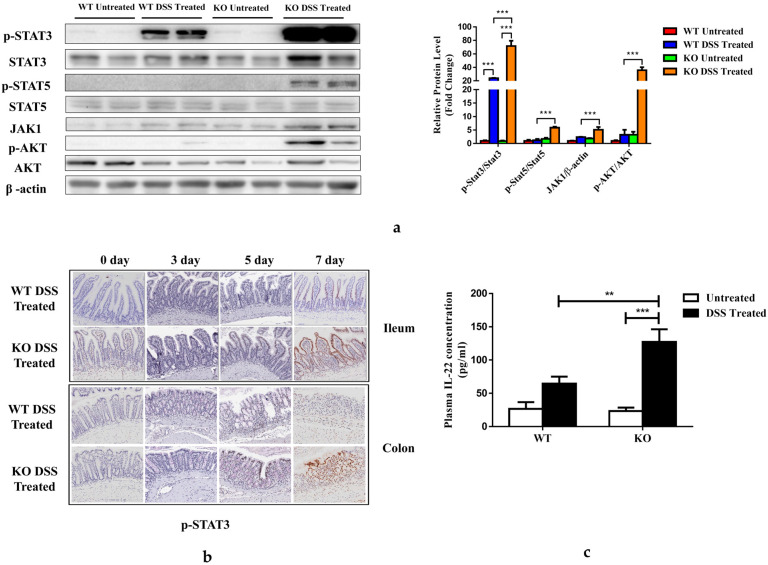
DSS treatment enhances mucosal expression of phospho-STAT3 in FGF21 KO mice. (**a**) Distal colon lysates were prepared and analyzed for expression and phosphorylation of indicated proteins (left panel); protein band intensity was quantified by densitometry analysis (right panel). (**b**) Immunohistochemical analysis of colon tissue phophos-Stat3 (p-Stat3). (**c**) Plasma IL-22 concentration. Data presented indicate the mean ± SEM. (**a**,**b**) Significance was determined using a two-way ANOVA, followed by Tukey’s multiple comparisons test (** *p* < 0.01 and *** *p* < 0.001).

Signal transducers and activators of transcription (STATs) mediate cytokine signaling and are important for the development of IBD [34]. We found that the 7-day DSS treatment increased STAT3 phosphorylation in the colon tissues of WT mice, and this activation was much more pronounced in the KO mice. DSS-induced STAT5 phosphorylation was undetectable in the colons of WT mice but was significantly enhanced in the KO mice (Figure 6a). Similarly, DSS treatment increased JAK expression and AKT phosphorylation in colon tissues only in the KO mice (Figure 6a). Previous studies indicated that the activation of STAT3 performs different functions in the epithelial cells and macrophages in IBD [35]. IL-6-mediated macrophage STAT3 phosphorylation is pathogenic [36], while interleukin-22 (IL-22)-mediated epithelial cell STAT3 phosphorylation is regulatory [37]. An immunohistochemical analysis of the colon and ileal tissues revealed that p-STAT3 was detected in the WT mice treated with DSS for 7 days. However, in the FGF21 KO mice, p-STAT3 staining was detected earlier, on day 5 after DSS administration, and was significantly increased on day 7. Most importantly, the expression of p-STAT3 was mainly observed in the epithelial cells, implying that the increased STAT3 activation might be protective (Figure 6b and Figure S2), given that DSS treatment markedly elevated plasma IL-6 concentration in the WT mice but not in the KO mice (Figure 3a). Importantly, DSS treatment had no effect on plasma IL-22 levels in the WT mice but significantly increased them in the KO mice (Figure 6c). Taken together, FGF21 depletion may suppress DSS-induced macrophage STAT3 activation by decreasing IL-6 production but may promote epithelial STAT3 activation by increasing the expression of IL-22. 

### 3.7. FGF21 Deficiency Decreases Colonic SOCS2/3 Expression

**Figure 7:**
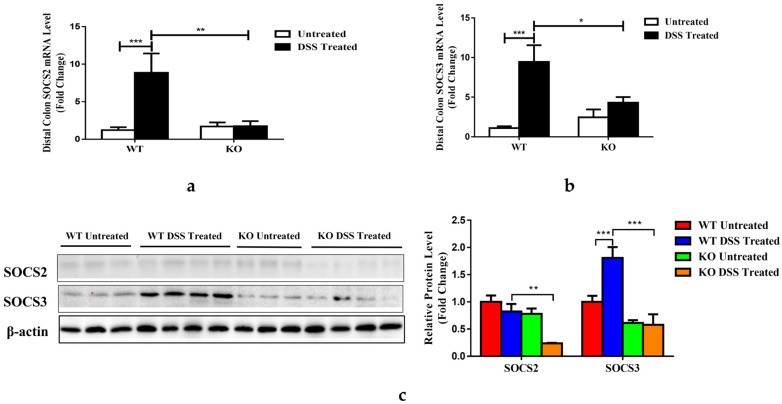
FGF21 regulates STAT3 activity through the SCOS2 and SCOS3 signaling pathway. SOCS2 (**a**) and SOCS3 (**b**) mRNA were measured via qRT-PCR in the distal colon. (**c**) Distal colon protein levels of SOCS2 and SOCS3 were analyzed by Western blotting (left panel). Protein band intensity was quantified by densitometry analysis, and β-actin levels were used as loading controls (right panel). Data presented indicate the mean ± SEM. (**a**–**c**) Significance was determined using a two-way ANOVA, followed by Tukey’s multiple comparisons test (* *p* < 0.05, ** *p* < 0.01 and *** *p* < 0.001).

Previous research showed that the suppressor of SOCS2 was elevated in FGF21 transgenic mice, and this was associated with a decrease in STAT5 phosphorylation [38]. We investigated the mechanisms by which FGF depletion affected SOCS2/3 signaling. As shown in Figure 7a,b, the DSS treatment significantly increased colonic SOCS2 and SOCS3 mRNA expression in the WT mice but not in the KO mice. An immunoblot analysis showed that DSS induced a robust increase in colonic SOCS3 protein levels but not SOCS2 in the DSS-treated WT mice (Figure 7c). However, both SCOS2 and SOCS3 protein levels were considerably lower in the KO mice compared to the WT mice treated with DSS. 

## 4. Discussion

FGF21 functions as an endocrine hormone and is produced mainly in the metabolic organs, such as the liver, adipose tissues, and muscles. It functions systemically by binding to FGFRs and KLB [9]. Previous studies have demonstrated that FGFRs and KLB are abundantly expressed in intestinal tissues, suggesting that FGF21 may be involved in intestinal pathophysiology. In this study, we discovered that FGF21 deficiency protected against DSS-induced colitis through epithelial IL-22-STAT3-mediated signaling.

FGF21 is important in the regulation of energy homeostasis in both preclinical and clinical studies. Consequently, utilizing the metabolic effects of FGF21 to treat MetS has received increasing attention. It has been shown that FGF21 is induced by multiple stimuli, including high-fat-diet feeding and alcohol consumption [13,39,40,41], which likely serve as adaptive responses to limit the detrimental effects of the stimuli. Recent studies showed that LPS-induced inflammation resulted in the increased expression of FGF21 [16], and colitis patients had higher levels of plasma FGF21 [7,20]. As inflammation is a hallmark of IBD, we initially hypothesized that lacking FGF21 might exacerbate IBD. To our surprise, FGF21-deficient mice had less body weight loss, less colon length shortening, and reduced inflammation compared to WT mice treated with DSS, suggesting that FGF21 depletion protected against DSS-induced colitis in mice. It is noteworthy that the administration of DSS brought about a marked elevation in MCP-1 expression in both WT and KO mice. While plasma MCP-1 level was significantly suppressed in the KO DSS-treated mice, the colon culture media level and colonic mRNA level were insignificantly decreased. IBD is a systemic inflammatory related disease. The discrepancy could be explained by the local and systemic regulation. 

An important finding in this study was the remarkable phosphorylation of STAT3 in FGF21 KO mice treated with DSS. STAT3 plays a critical role in colitis pathology, and its activation is mediated by multiple cytokines such as IL-6. IL-6 binds its receptors and recruits JAK to phosphorylate signal-transducing beta-receptor (gp130), which interacts with IL-6 α-receptors, thereby activating JAK/STAT3 and Phosphoinositide 3-kinase (PI3K)/AKT/mammalian target of rapamycin (mTOR) pathways [42]. AKT, also known as threonine/serine kinase, plays key roles in multiple signaling pathways involving apoptosis, cell migration, cell survival, cell proliferation and differentiation, and transcription [43]. Early studies revealed that the PI3K-AKT signaling pathway is critical for the progression of colitis via the activation of inflammatory signaling by regulating NF-κB in macrophages [44,45]. Additionally, the PI3K/AKT pathway also plays an important role in the regulation of intestinal epithelial proliferation, survival, and wound healing [46,47]. Furthermore, the elevated expression of IL-6 was reported to be positively associated with increased disease activity in CD and UC patients [48,49]. However, the protective effects of FGF21 KO against DSS-induced colitis and the colonic phosphorylation of STAT3 cannot be explained by the IL-6 regulation since IL-6 expression was reduced in the KO mice. Previous studies showed that the phosphorylation of STAT3 is also mediated by IL-22 signaling [50]. IL-22 belongs to the IL-10 family of cytokines and plays an anti-inflammatory role in the intestine. The loss of IL-22 resulted in severe colitis, which was rescued by increasing the expression of IL-22 [51,52,53]. The protective effects of IL-22 in colitis were dependent on epithelial STAT3 activation [35]. In contrast to IL-6, DSS induced a robust increase in IL-22 expression in the KO mice, suggesting that the activation of STAT3 is mediated by IL-22 signaling. 

In addition to the elevation of IL-22 expression, our data also suggest that SOCS2/3 were involved in the regulation of epithelial STAT3 activation. It was reported that the overexpression of FGF21 increased SOCS2 expression [38], which suppressed STAT3 activation. FGF21 depletion eliminated DSS-induced increases in SOCS3 mRNA and protein expression and decreased the expression of SOCS2 protein and mRNA. These data suggest that SCOS2/3 reduction was critical to the protective effects of FGF21 deficiency on DSS-induced colitis. 

Paneth cells, which reside in the small intestinal crypts, secrete antimicrobial peptides and maintain homeostasis of the gut microbiota [54]. Goblet cells in the epithelial lining secrete mucin, which aggregates to form the mucus layer, acting as a physical barrier between the intestinal lumen and the epithelial lining [55]. Defective goblet cells and Paneth cells have been associated with IBD in humans [56,57,58]. Interestingly, a previous study showed that IL-22 delivery enhanced STAT3 activation in the colon epithelial cells and induced restitution of goblet cells [52]. Paneth cells and epithelial stem cells can form a niche to maintain epithelial regeneration and host–microbe interactions [59,60]. This niche has been reported to be regulated by the IL-22-pSTAT3 pathway [61]. Therefore, FGF21 KO mice may maintain goblet and Paneth cell homeostasis in colitis through the activation of IL-22-STAT3 signaling [62].

Our study has limitations. It is not clear how FGF21 deletion causes an increase in IL-22 and a decrease in IL-6 in mice with acute colitis. Previous studies demonstrated that IL-22 protects against myocardial infarction by stimulating hepatic FGF21 expression [62,63]. Whether FGF21 induction causes a feedback inhibition of IL-22 requires further investigation. DSS-induced colitis is characterized by a changed gut microbiota homeostasis (dysbiosis), which is associated with an increased inflammatory response and intestinal barrier injury [64,65]. A gut-dysbiosis-associated decrease in the production of short-chain fatty acids (SCFAs) has also been reported. Gut-microbiome-derived SCFAs upregulate IL-22 production by promoting hypoxia-inducible factor 1α (HIF-1α) expression, and FGF21 is likely an inhibitor of HIF1α [36,63]. It is thus possible that the inflammatory response during colitis-induced FGF21 expression inhibits intestinal HIF1α expression, leading to a decrease in IL-22 and therefore exacerbating the expression of inflammatory IL-6 production. The deletion of FGF21 attenuates the inhibitory effects and promotes IL-22 expression. 

## 5. Conclusions

Overall, we demonstrated that endogenous FGF21 was increased in DSS-induced acute colitis, which contributed to the progression of the disease. FGF21 deletion protected mice from DSS-induced acute colitis by increasing IL-22 expression-mediated epithelial STAT3 activation, which decreased intestinal inflammation and maintained intestinal goblet cell and Paneth cell homeostasis. Although the effects of exogenous FGF21 treatment on acute and chronic colitis and colitis recovery and remission were not clearly investigated due to the complex effects of FGF21, we propose that targeting the FGF21 signaling pathway could be a promising strategy for treating IBD. Further investigation of the precise role and mechanism of FGF21 in IBD may provide a clear direction for developing therapeutic treatment. 

## Figures and Tables

**Table 1:** Primer sequences used for qRT-PCR.

Name	Sequences (Forward/Reverse 5′–3′)	
CXCL10	GGTCTGAGTGGGACTCAAGG	GTGGCAATGATCTCAACACG
FGF21	CCTCTAGGTTTCTTTGCCAACAG	AAGCTGCAGGCCTCAGGAT
IL-1β	TTCATCTTTGAAGAAGAGCCCAT	TCGGAGCCTGTAGTGCAGTT
IL-6	TGGAAATGAGAAAAGAGTTGTGC	CCAGTTTGGTAGCATCCATCA
TNF-α	CACCACCATCAAGGACTCAA	AGGCAACCTGACCACTCTCC
MCP-1	GGCTCAGCCAGATGCAGT	GAGCTTGGTGACAAAAACTACAG
Socs2	TCCAGATGTGCAAGGATAAACG	AGGTACAGGTGAACAGTCCCATT
Socs3	ATTTCGCTTCGGGACTAGCTC	AGCTGTCGCGGATAAGAAAGG
18s	CTAACCCGTTGAACCCCATT	CCATCCAATCGGTAGTAGCG

## Data Availability

Data sharing is not applicable to this article.

## Supplementary Materials

The following supporting information can be downloaded at: https://www.mdpi.com/article/10.3390/nu15092086/s1, Figure S1. FGF21 KO mice display enhances intestinal epithelial cell proliferation responses upon DSS treatment. Representative BrdU staining of colonic tissues; Figure S2. DSS treatment enhances mucosal expression of phosphor-Stat3 in FGF21 KO mice. WT and FGF21 KO mice were either untreated or treated with 2.5% DSS for 7 days. Immunohistochemical analysis of phophos-Stat3 (p-Stat3) of the colonic and ileal tissue.
