# Supplementary material for: FGF21 Depletion Attenuates Colitis through Intestinal Epithelial IL-22-STAT3 Activation in Mice
Source: Nutrients. 2023 Apr 26;15(9):2086. doi: 10.3390/nu15092086 (PMC10181108; doi:10.3390/nu15092086)
Supplement: Supplementary file 1 [file nutrients-15-02086-s001.zip › nutrients-2335296-supplementary.pdf]

*Article*

# FGF21 Depletion Attenuates Colitis through Intestinal Epithelial IL-22-STAT3 Activation in Mice

**Liming Liu [1,2], Fengyuan Li [2,3], Tuo Shao [2], Lihua Zhang [2], Jiyeon Lee [2,3], Gerald Dryden [2], Craig J. McClain [2,3,4,5,6], Cuiqing Zhao [1,2,*] and Wenke Feng [2,3,4,5,*]**

1 College of Animal Science and Technology, Jilin Agricultural Science and Technology University, Jilin 132101, China; aliuliming1984@126.com
2 Department of Medicine, University of Louisville, Louisville, KY 40202, USA; fengyuan.li@louisville.edu (F.L.); tuoshao89@gmail.com (T.S.); lihua.zhang@louisville.edu (L.Z.); jiyeon.lee@louisville.edu (J.L.); gerald.dryden@louisville.edu (G.D.); craig.mcclain@louisville.edu (C.J.M.)
3 Department of Pharmacology &Toxicology, University of Louisville, Louisville, KY 40202, USA
4 Hepatobiology & Toxicology Center, University of Louisville, Louisville, KY 40202, USA
5 Alcohol Research Center, University of Louisville, Louisville, KY 40202, USA
6 Robley Rex VA Medical Center, Louisville, KY 40206, USA
* Correspondence: yuqing53@163.com (C.Z.); wenke.feng@louisville.edu (W.F.); Tel.: +86-0432-6470-3108 (C.Z.); +1-502-852-2920 (W.F.)

**Supplementary Figures**

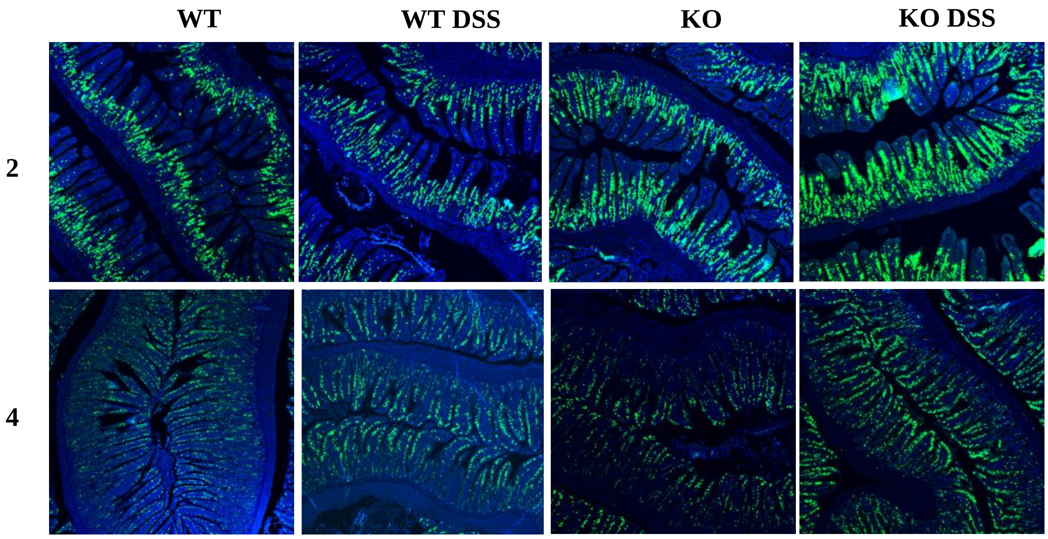


**Figure S1.** FGF21 KO mice display enhances intestinal epithelial cell proliferation responses upon DSS treatment. Representative BrdU staining of colonic tissues.

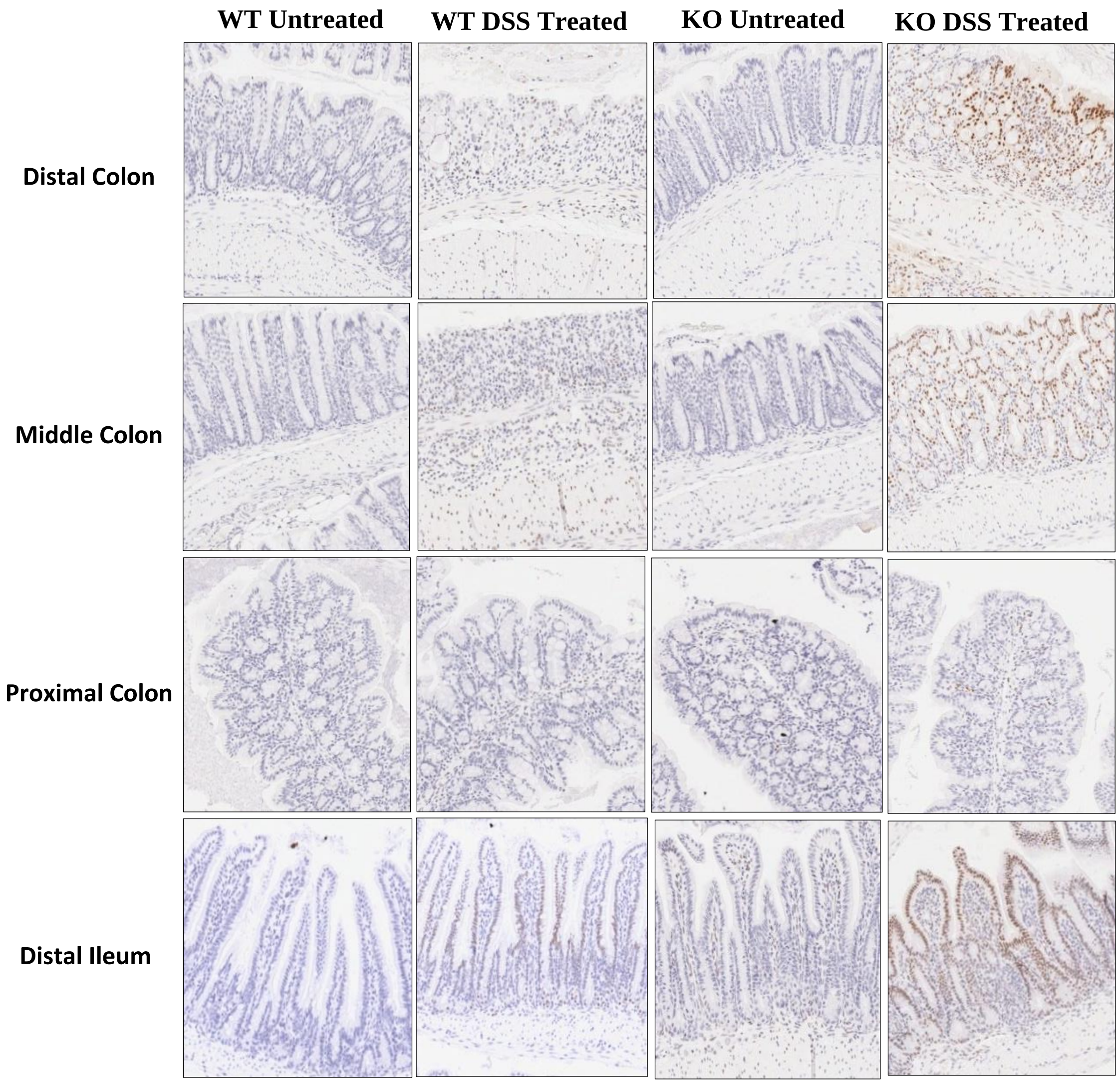


**Figure S2.** DSS treatment enhances mucosal expression of phosphor-Stat3 in FGF21 KO mice. WT and FGF21 KO mice were either untreated or treated with 2.5% DSS for 7 days. (A) Immunohistochemical analysis of phophos-Stat3 (p-Stat3) of the colonic and ileal tissue.
